# Pulsed-field ablation vs. thermal ablation for paroxysmal atrial fibrillation: a systematic review and meta-analysis of randomized controlled trials

**DOI:** 10.1093/europace/euaf179

**Published:** 2025-08-14

**Authors:** Patrick Badertscher, Jeanne Du Fay de Lavallaz, Tobias Reichlin, Laurent Roten, Thomas Kueffer, Fabian Jordan, Sven Knecht, Felix Mahfoud, Christian Sticherling, Michael Kühne

**Affiliations:** University Hospital of Basel, University of Basel, Petersgraben 4, Basel 4031, Switzerland; Cardiovascular Research Institute Basel, Basel 4031, Switzerland; University Hospital of Basel, University of Basel, Petersgraben 4, Basel 4031, Switzerland; Cardiovascular Research Institute Basel, Basel 4031, Switzerland; Inselspital Bern, University of Bern, Bern, Switzerland; Inselspital Bern, University of Bern, Bern, Switzerland; Inselspital Bern, University of Bern, Bern, Switzerland; University Hospital of Basel, University of Basel, Petersgraben 4, Basel 4031, Switzerland; Cardiovascular Research Institute Basel, Basel 4031, Switzerland; University Hospital of Basel, University of Basel, Petersgraben 4, Basel 4031, Switzerland; Cardiovascular Research Institute Basel, Basel 4031, Switzerland; University Hospital of Basel, University of Basel, Petersgraben 4, Basel 4031, Switzerland; Cardiovascular Research Institute Basel, Basel 4031, Switzerland; University Hospital of Basel, University of Basel, Petersgraben 4, Basel 4031, Switzerland; Cardiovascular Research Institute Basel, Basel 4031, Switzerland; University Hospital of Basel, University of Basel, Petersgraben 4, Basel 4031, Switzerland; Cardiovascular Research Institute Basel, Basel 4031, Switzerland

**Keywords:** Atrial fibrillation, Pulmonary vein isolation, Pulsed-field ablation, Thermal ablation

Radiofrequency and cryoablation have been the standard energy sources for pulmonary vein isolation (PVI) in patients with atrial fibrillation (AF), whereas pulsed-field ablation (PFA) has emerged as a treatment alternative.^1,2^ Large observational studies have suggested that PFA is both safe and effective.^3^ Two randomized trials have now compared PFA with thermal energy sources. The ADVENT trial^4^ and the SINGLE SHOT CHAMPION trial^5^ randomized participants with paroxysmal AF to PFA or thermal energy sources. The objective of this study-level meta-analysis was to systematically review, synthesize, and appraise published randomized trials comparing the efficacy and safety of PFA with thermal energy sources for the treatment of paroxysmal AF.

A structured systematic search of PubMed, MEDLINE, and Embase was conducted for randomized controlled trials (RCTs) published between 2018 and 2025 using the following keywords: pulsed-field, cryoballoon, thermal, and radiofrequency. Eligible studies included RCTs reporting on the efficacy and/or safety of PVI using PFA compared with thermal energy in patients with paroxysmal AF. The primary efficacy endpoint was the absence of atrial arrhythmia recurrence through 3 and 12 months following ablation. Statistical analyses were conducted employing random-effects models for continuous and binary outcomes. The variance between studies was calculated using the DerSimonian–Laird estimator, and the confidence interval of the tau-squared was calculated by using the Jackson method.

**Figure 1:**
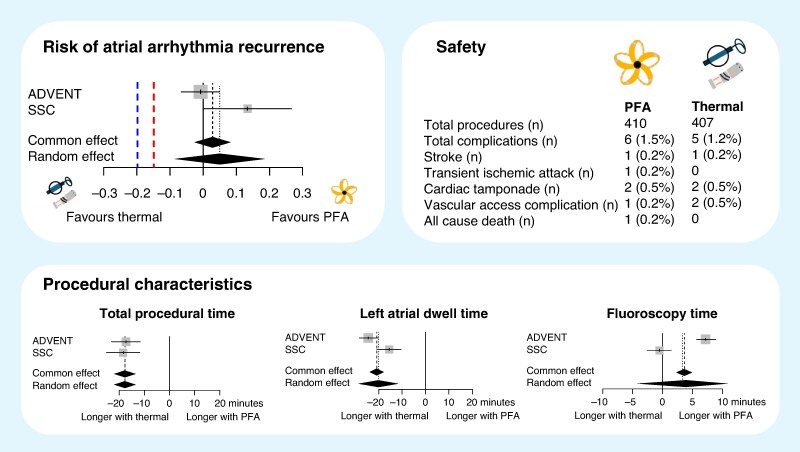
Summary of randomized trial evidence (ADVENT and SINGLE SHOT CHAMPION) comparing pulsed-field versus thermal ablation for paroxysmal atrial fibrillation.

The systematic literature review, completed on 8 April 2025, yielded 351 abstracts, of which two RCTs met the criteria, encompassing 817 patients. Four hundred ten patients were ablated with PFA and 407 with thermal energy. The weighted mean age was 62 years, and the mean proportion of women was 33%. Both RCTs used a non-inferiority design, and the outcome of absence of atrial tachyarrhythmia recurrence between 3 and 12 months. Pulsed-field ablation was non-inferior to thermal energy for the primary outcome, although superiority was not achieved [risk difference (RD) of a random-effects model 0.05 95% CI (−0.09–0.19)]. Other efficacy outcomes (need for cardioversion after 3 months, antiarrhythmic treatment, and redo ablation) showed no difference between both ablation energies (*Figure 1*). In both studies, PFA was associated with a significantly shorter procedural duration [standardized mean difference (SMD) −17.7 min, *P* < 0.001] and significantly shorter left atrial dwell time (SMD −19.9 min, *P* < 0.001) with no difference in fluoroscopy duration (SMD 3.36 min, *P* = 0.383). Serious complications up to 30 days (death, phrenic nerve palsy, stroke/transient ischaemic attack, cardiac tamponade, vascular access complication, or extraoesophageal fistula) were rare and occurred in 6/410 (1.46%) of the PFA patients and in 5/407 (1.23%) of the thermal energy patients.

In this meta-analysis of two contemporary RCTs, PFA showed non-inferiority as compared with thermal ablation with a comparable risk profile and was associated with shorter procedure times. The following key differences between the two RCT are noteworthy: the ADVENT trial used intermittent Holter monitoring, prone to underreporting of atrial arrhythmia recurrences,^6^ while SINGLE SHOT CHAMPION employed implantable cardiac monitors. Operators in ADVENT were new to PFA, while at least 6 months experience with PFA was present in SINGLE SHOT CHAMPION. The ADVENT trial included both cryoballoon and radiofrequency ablation as comparators, which are known to have different safety profiles, whereas the SINGLE SHOT CHAMPION trial focused exclusively on cryoballoon ablation. While in ADVENT no information regarding atrial arrhythmia recurrence during the blanking period was provided, in SINGLE SHOT CHAMPION a significantly lower recurrence rate was found for PFA.

Taken together, these differences limit direct comparability between the two trials and reduce the clinical interpretability of pooled estimates from this meta-analysis. In addition, the inclusion of only two randomized trials with the rather small total sample size of 817 patients limits the power to detect significant differences in long-term outcomes or rare adverse events. A prior meta-analysis of predominantly observational studies encompassing 2255 patients confirmed the favourable safety profile of PFA, with lower rates of oesophageal injury and phrenic nerve palsy, but higher rates of pericardial tamponade.^7^ Similarly, Vetta *et al.*^8^ reported lower overall complication rates with PFA compared with cryoballoon ablation.

While the SINGLE SHOT CHAMPION trial included hierarchical testing for superiority following non-inferiority, our meta-analysis, based on study-level data from both trials, does not allow definitive conclusions regarding superiority. Accordingly, our findings should be interpreted within the broader context of non-inferiority-focused evidence. Furthermore, as both trials used the Farapulse system, the findings of this meta-analysis cannot be generalized to other pulsed-field ablation platforms.

In conclusion, this meta-analysis shows that PFA is a non-inferior and more efficient alternative to thermal ablation for patients with paroxysmal AF but also emphasizes the importance of operator experience and monitoring techniques in determining the success rate of PFA. Future research should focus on testing superiority endpoints, include larger populations, extend follow-up durations, and incorporate standardized rhythm monitoring and include non-paroxysmal AF patients to establish the role of PFA in the management of AF.
